# Prediction of the Tensile Response of Carbon Black Filled Rubber Blends by Artificial Neural Network

**DOI:** 10.3390/polym10060644

**Published:** 2018-06-09

**Authors:** Ivan Kopal, Ivan Labaj, Marta Harničárová, Jan Valíček, Dušan Hrubý

**Affiliations:** 1 Faculty of Industrial Technologies in Púchov, Alexander Dubček University of Trenčín, Ivana Krasku 491/30, 020 01 Púchov, Slovakia; ivan.labaj@fpt.tnuni.sk; 2 Faculty of Engineering, Slovak University of Agriculture in Nitra, Trieda Andreja Hlinku 609/2, 949 76 Nitra, Slovakia, marta.harnicarova@uniag.sk (M.H.), jan.valicek@uniag.sk (J.V.), dusan.hruby@uniag.sk (D.H.); 3 Department of Mechanical Engineering, Faculty of Technology, Institute of Technology and Business in České Budějovice, Okružní 10, 370 01 České Budějovice, Czech Republic

**Keywords:** carbon black filled rubber blends, mechanical properties and characteristics, tensile tests of polymers, stress-strain curves, artificial neural network

## Abstract

The precise experimental estimation of mechanical properties of rubber blends can be a very costly and time-consuming process. The present work explores the possibilities of increasing its efficiency by using artificial neural networks to study the mechanical behavior of these widely used materials. A multilayer feed-forward back-propagation artificial neural network model, with a strain and the carbon black content as input parameters and stress as an output parameter, has been developed to predict the uniaxial tensile response of vulcanized natural rubber blends with different contents of carbon black in the form of engineering stress-strain curves. A novel procedure has been created for the simulation of the optimized artificial neural network model with input datasets generated by a regression model of an experimental dependence of tensile strain-at-break on the carbon black content in the investigated blends. Errors of the prediction of experimental stress-strain curves, as well as of tensile strain-at-break, tensile stress-at-break and M100 tensile modulus were estimated for all simulated stress-strain curves. The present study demonstrated that the performance of a developed neural network model to predict the stress-strain curves of rubber blends with different contents of carbon black is also exceptionally high in the case of a network that had never learned the input data, which makes it a suitable tool for extensive use in practice.

## 1. Introduction

Typical rubber blends (RB), which are now commonly used in an extensive range of practical applications [1], contain over ten different ingredients [2] that are added to affect their vulcanization, improve processability, enhance several physical properties, and prevent long-term deterioration of the final rubber-based products [3]. To obtain the desired mechanical properties, they need to be reinforced by suitable fillers [4]. In recent times, carbon black (CB) is the most widely used material for such reinforcement [5]. Each CB filled RB consists of a cross-linked rubber matrix with CB particles, leading to an increase of stiffness properties [6] and hysteresis of the resulting composite [7], as well as to its strain rate dependence [8]. Predicting the relationship between the final mechanical properties of CB reinforced RB, and the content of CB filler in the RB matrix plays a key role in the efficient design of the composition of the RB for various applications [9], therefore, it is in the interest of our research.

Tensile properties, reflecting the mechanical response of the material to external forces applied in tension, are determined by tensile testing [10]. In the course of tensile testing, a standard carefully prepared test specimen is loaded in a controlled uniaxial tension manner, the applied load and the elongation (displacement) of the test specimen over some distance are measured and a load versus elongation curve, which is subsequently converted into an engineering stress versus engineering strain curve, is constructed. Results of tensile tests make it possible to identify different tensile properties and mechanical characteristics of the tested material, such as tensile and yield strength, yield point, elongation, stress and strain at break, modulus of elasticity, elastic and proportional limit, as well as several others [11]. In general, stress-strain curves of some polymeric materials, such as rubbers, elastomers etc., have a rather complex non-linear character in its entirety, what complicates their analytical description using classical, physically based mathematical models closely agreeing with the experimental data up to the point of the test specimen rupture [12]. Moreover, the properties of these materials change significantly with temperature and strain rate, making their modelling even more difficult, compared with other traditional engineering materials. Usually, these structures cannot be defined analytically because of different physical and material nonlinearities or their complex geometry [13]. Nowadays, there are several classes of phenomenological approaches to developing a suitable constitutive stress-strain relation that can fit experimental results for rubber-like or hyperelastic materials. A detailed overview is given in papers [14]. The most significant differences in approach to such modelling can be found in particular between constitutive models focusing on the rate of the independent softening behavior of the virgin hyperelastic material [15], models describing its instantaneous response at high strain rates [16], as well as models relating the repeatable dynamic behavior of the non-virgin material under cyclic loading [17]. However, all hyperelastic materials can be characterized by a stored or strain energy function, in which the strain energy density is expressed either in terms of principal stretch ratios or in terms of deviatoric strain invariants and volume ratio [18]. The coefficients occurring in the strain energy function should be determined by experimental data of uniaxial and biaxial tensile as well as shear tests. The ultimate problem is to determine the strain energy function for providing a good fit with a number of sets of experimental data. The review [19] introduces the most commonly used constitutive models, such as the elastic and hyperelastic model, polynomial and reduced polynomial model, the Ogden model, the Mooney—Rivlin model and others. Progress in the field of machine learning in recent years has shown that an efficient way to completely characterize the strain energy function of hyperelastic materials goes through the use of artificial neural networks (ANNs) [20]. Unlike the model-driven approaches of the classical mathematical methods, ANN models belong to the class of data-driven approaches, which can directly cover all the complicating effects of the problem under investigation without the need to understand their physical nature and the mutual relationships between them based on learning from representative experimental data [21]. 

In this work, an ANN approach was used to create a powerful predictive model for stress-strain curves of RB with different contents of CB as a whole over a wide range of CB/RB ratio. In fact, producers of rubber-based products typically modify their mechanical properties according to customer requirements only by adding different amounts of CB to RB, which is typically used to produce a broader range of products. Any such modification requires time-consuming and expensive tensile tests that can be successfully replaced by a simulation of stress stress-strain curves using a reliable well-trained ANN.

Artificial neural networks represent intelligent, highly sophisticated, biologically inspired analytical tools that are especially effective in solving such problems where the correlations between the dependent and independent data are well known, but their precise description by classical mathematical methods is too complicated, too simplified, or impossible [22]. The main advantage of ANNs is their intelligence; i.e., their ability to learn through the patterns presented in the form of input and corresponding output parameters, which can be generated from experimental results [23]. During learning from the patterns, each ANN automatically repeats the mapping of the relationship between the input and output parameters that have been inserted into it until it finds a sufficiently reliable model of the analyzed experimental data. Since the acquired knowledge from this mapping is (similarly as in the human brain) stored in the ANN structure in a distributed fashion in the form of synaptic strengths (weights) and thresholds (biases), it can be generalized and then used in the proper prediction of relationships between the input and output parameters that were not part of carefully selected learning or training pattern describing the investigated problem [24]. At present, the most commonly used type of ANNs is a multilayer feed-forward back-propagation ANN (FF-BP-ANN) because of its structural simplicity, fast learning ability, and high predictive performance [25]).

The FF-BP-ANNs are nonparametric powerful mathematical models specially designed to find however complicated nonlinear relationships between the specified input and output patterns generated from the experimental results. They are incredibly efficient especially in an approximation of any continuous function with a finite number of discontinuities by mapping the relationships between the input (independent) and output (dependent) datasets [26].

Similarly, as all other types of ANNs, the FF-BP-ANNs also consist of artificial neurons, which are their elementary building and computation units [27]. These units are, however, ordered into one input, one output, and at least one hidden layer located between the input and output layer (multilayer), with an input set, output set, and hidden set of neurons, as well as with suitably chosen transfer or activation functions representing the transformation functions of the input and of the output signals of individual neurons [28]. The information brought into the input layer propagates through all the hidden layers of the network in a forward direction to its output layer. Each neuron can receive input signals from the lower layer neurons or directly from the environment, to process them, to generate an output signal and send it to the neurons on the higher layer only (feed-forward). Each neuron of each layer is connected to each neuron of the neighboring layer (fully connected ANN), but there is no connection between neurons of the same layer or between the neurons, which are not in the successive layers. The synaptic strength of the connection between neurons and between the neurons and environment is evaluated by a weight factor representing the probability of data transmission along a path connection. The overall input signal to each neuron represents the weighted sum of the outputs of all neurons connected to it and the bias connecting the given neuron to the environment. The number of hidden layers and the number of hidden neurons, or the topology of the FF-BP-ANN, determine its capacity. It has been proven that even the FF-BP-ANNs with only one hidden layer and non-linear hidden transfer functions can work as a universal approximator that can approximate any linear and non-linear differentiable functional dependence with many variables [29].

The knowledge about the relations between the input and output data is stored in the weights of the synaptic connections between particular neurons and in the biases. The values of weights and biases are updated iteratively in the process of supervised learning of the network with a set of representative values of input-output data patterns minimize the difference between the actual output of the network and the desired output for each pattern on the training dataset [30]. In this minimization process, based on a gradient search with the least sum squared optimality criterion of errors between the predicted and the desired values (network error), the weights of all the connecting neurons and biases are adjusted until the desired error level is achieved or the maximum number of learning iteration is reached. At every iteration of the learning process, the error of the network is computed. The error signal is then propagated from the output layer to the input layer across all the network neurons (error back-propagation), and according to the size of the backward propagated error, the individual weights of the neurons and that of the biases are appropriately modified [31]. The Levenberg—Marquardt back-propagation minimization algorithm has been used to adjust these weights in the presented work precisely as just as in over 80% of all applications using the multilayer FF-BP-ANNs, and it will be described later in a more convenient place [32].

The concept of the multilayer FF-BP-ANN with the Levenberg—Marquardt back-propagation minimization algorithm is the design of a reliable description and reliable prediction of experimental functional dependence of the input and output data mechanism, or a concept of the solution of the problem of fitting the experimental data with the ANNs, relating to polymers, their composites, and nanocomposites. It is discussed in detail in a number of survey publications; e.g., in the papers [33,34,35], or in our earlier work [36] devoted to ANN modelling and the prediction of experimental results of a dynamic mechanical analysis of thermoplastic polyurethane over a wide range of temperature. It has been shown that an optimized FF-BP-ANN model is a much more effective and efficient alternative to the unified statistical stiffness-temperature model for thermoplastic polymers based on the Weibull distribution of the secondary bonds rupture in polymeric materials in their cyclical mechanical stresses. In [37], there is a detailed description of the modelling process of the mechanical properties of CB reinforced RB using machine learning techniques. A powerful ANN model intended to predict the modulus at 100% deformation, shore A hardness and tensile strength, three relevant physical attributes of RB, was built and subsequently evaluated in this paper. A FF-BP-ANN, consisting of abrasion and the six mechanical characteristics of SBR-based rubber (shore A hardness, stress at 100%, stress at 300%, tensile strength, elongation at break, and tear strength) of twenty sets of SBR-based composites, was established and three sets of validation data were used to predict the abrasion of SBR-based rubber to verify the reliability of the ANN in the work [38]. The paper [39] is devoted to the prediction of rubber mechanical properties, such as tensile strength as well as modulus at 100% deformation, in an aged and non-aged state using the FF-BP-ANN. In the study [40], multilayer FF-BP-ANN, an Elman network, and a generalized regression ANN were used for modelling the cure curves of rubber blends at different temperatures. The ability of selected ANN architectures on predicting optimum cure times of 11 different rubber compounds in a model tire was studied. It has been concluded that ANNs can be used as a very powerful and simple alternative technique for the prediction of an optimum cure time to equivalent cure concept, which is traditionally used in the rubber and tire industries. In the work [41], the effective and efficient ANN architecture was found to optimize tire design before an expensive finite element analysis used to confirm the predicted tire performance. Generally speaking, there are few publications about the use of ANNs in the area of polymeric materials or rubbers up to now. However, the available literature at least covers various topics, from fatigue prediction to wear simulation, and from monitoring of the manufacturing process to the analysis of the curing [42]. As has already been mentioned, the present work deals with the predictive modelling of tensile stress-strain curves of RB with different contents of CB using the curve fitting of experimental data through a FF-BP-ANN. 

## 2. Materials and Methods

The composition of RB with different contents of CB was the following: 100 phr of standard Malaysian natural rubber grade SMR 10 (Lee Rubber, Kuala Lumpur, Malaysia); 15, 25, 40, 60, and 85 phr of carbon black grade N339 (Makrochem, Lublin, Poland); 5 phr of zinc oxide; 2 phr of stearic acid; 2.5 phr of sulphur and 1.5 phr of N-tert-butyl-2-benzothiazole sulphonamide (all these additives were supplied by Bayer, Kuala Lumpur, Malaysia). The range of investigated CB content from 15 phr to 85 phr was determined by the resulting mechanical properties and mechanical characteristics of the RB.

The mixing of all chemicals was carried out using a two-stage method in a universal torque rheometer Brabender Plastograph EC Plus (Brabender, Duisburg, Germany) with a mixing speed of 50 ± 1 rpm at a temperature of 90 ± 1°C. After the mixing process, the mixture was homogenized in a laboratory two-roll mill LaboWalz (Vogt, Berlin, Germany) for three minutes and then allowed to stand for 24 h at room temperature. An optimum vulcanization time *t*_c(90)_ was measured using a PRPA 2000 rheometer (Alfa Technologies, Akron, OH, USA) individually for each RB with different CB content. The prepared compounds were vulcanized into 2 ± 0.02 mm thick plates in a Fontijne LabEcon 600 vulcanization press machine *(*Fontijne Presses, Vlaardingen, Netherlands) at a temperature of 150 ± 1 °C with respective vulcanization time *t*_c(90)_ for each CB content in the RB and at a pressure of 50.8 ± 0.1 psi. The vulcanized blends could stand for 24 h. From the vulcanized RB plates, the test specimens in the shape of dumb-bell type II (according to ISO 37 or ASTM D 412) were cut using an Instron cutter machine (Instron, Norwood, MA, USA). The uniaxial tensile testing of the test specimens was performed using a computer-controlled tensile tester Shimadzu AG-X Plus (Shimadzu, Tokyo, Japan) with two pneumatic clamping jaws with a reciprocal movement speed of 500 ± 5 mm·min^−1^. Tensile tests were performed at room temperature on three samples for each CB content in the RB. The average engineering stress-strain curves were constructed from the acquired experimental force versus displacement data.

### 2.1. ANN Structure and Architecture

For the prediction of the experimental results of the uniaxial tensile tests, a multilayer FF-BP-ANN model was developed. The structure of the ANN consists of one input, one hidden, and one output layer. The input data of the model are experimental values of the CB content *c* in the RB and values of engineering tensile strain *ε*(*c*) of the relevant test specimens, while the target data are the corresponding experimental values of engineering tensile stress *σ*(*ε*,*c*) at a given *c* and *ε*(*c*). The input layer of the network has two neurons, and the output layer has one neuron, which corresponds to the number of the input and output variables of the model. The number of hidden neurons was searched for in the process of optimizing the model with the use of trial and error method [43]. A hyperbolic tangent sigmoid transfer function *tansig*(*n*) between the input and hidden layer and a pure linear transfer function *purelin*(*m*) between the hidden and output layer were used, where *n* and *m* are the net input into the transfer function of the relevant ANN layer [44].

The role of the input layer is only to transport the input signal **p** to the hidden neurons. Each neuron of the hidden and output layer contains an input, weight, bias, summing junction, transfer function and output. Each element *i* of the input signal **p**, or of the input data matrix

(1) $$p\;=\;\left(\begin{array}{l} c_{11}\;c_{12}\;c_{13}\;\ldots \;c_{1R^{1}}\\ \varepsilon_{21}\;\varepsilon_{22}\;\varepsilon_{23}\;\ldots \;\varepsilon_{2R^{1}} \end{array}\right)\;,$$

is at the input to each hidden neuron multiplied by its weight *iw_ji_*^1,1^. These weighted inputs of individual *S*^1^ neurons *j* of the hidden layer are mutually summed in their summing junctions and the bias *b*^1^*_j_* of the current neuron *j*, representing a constant external input with a value of 1 multiplied by its weight, is added to the result [45].

The weighted sum of inputs and bias of each neuron *j* of the hidden layer generates the activation potential or the net input of the hidden neuron [46]

(2) $$n_{j}^{1}\;=\sum_{i\;=\;1}^{2R^{1}}iw_{ji}^{1,1}p_{i}+\;b_{j}^{1},\;j\;=\;1,\;2,\;3,\;\ldots \;,\;S^{1}$$

leading to its activation, which is then inserted into the non-linear transfer function *tansig* producing its output in the form

(3) $$a_{j}^{1}\;=\;f^{1}\left(n_{j}^{1}\right)\;=\;tansig\left(n_{j}^{1}\right)\;=\;\frac{e^{n_{j}^{1}}\;-\;e^{-n_{j}^{1}}}{e^{n_{j}^{1}}\;+\;e^{-n_{j}^{1}}}\;.$$

The total output of the hidden layer, consisting of *S*^1^ hidden neurons, can then be written in a matrix form as [47]

(4) $$a^{1}\;=\;f^{1}\left(IW^{1,1}p\;+\;b^{1}\right)$$

and it is then sent to the input of the output layer.

Subsequently, the net input of the output layer

(5) $$m_{k}^{2}\;=\;\sum_{j\;=\;1}^{S^{1}}lw_{k,j}^{2,1}a_{j}^{1}\;+\;b_{k}^{2},\;k\;=\;1$$

is inserted into the pure linear transfer function *purelin* which will generate its output signal

(6) $$a_{k}^{2}\;=\;f^{2}\left(m_{k}^{2}\right)\;=\;purelin\;\left(m_{k}^{2}\right)\;=\;m_{k}^{2},$$

or the total output signal of the output layer in the form

(7) $$a^{2}\;=\;f^{2}\left(LW^{2,1}a^{1}\;+\;b^{2}\right)\;,$$

and it is, therefore, possible to present the total output signal of the network **y** as:

(8) $$y\;=\;a^{2}\;=\;f^{2}\left(LW^{2,1}f^{1}\left(IW^{1,1}p\;+\;b^{1}\right)\;+\;b^{2}\right)\;.$$

**Figure 1:**
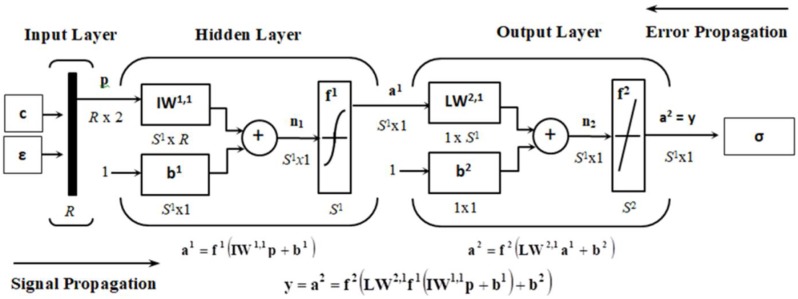
The scheme of multilayer feed-forward back-propagation artificial neural network for stress-strain curves prediction**.**

The upper indexes ^1^ and ^2^ as the hidden and output layer of the network are marked with superscripts ^1^ and ^2^, respectively, the subscript *_i_* denotes the elements of the input signal, the subscript *_j_* denotes the hidden layer neurons, and the subscript *_k_* represents a single neuron of the ANN output layer. The matrices and vectors are, in contrast to scalar values and functions, written in bold. The FF-BP-ANN architecture is designed as fully connected (Figure 1).

### 2.2. Training, Validation, and Testing of ANN

In general, an optimization of architecture, or of the topology of ANN model, is realized in the process of training, validation, and testing of the network. Its task is to find the neurons in the hidden layer, as well as ANN weights and biases, that best suits the needs of the problem to be solved. For this purpose, the input and target data of the network are divided into training, validation, and testing datasets in an appropriate ratio [48].

During supervised learning, the network gradually learns, using the training input-target patterns, how to discern the mutual interrelationships between the input and output tensile tests data in such a way that via a step-by-step modification of the weights **IW^1, 1^**, **LW^2, 1^** as well as the biases **b^1^**, **b^2^**, it minimizes the difference between the values *d_q_* of the vector of the desired data **d** and the *y_q_* values of the output vector **y** produced by the ANN [49]. The difference **δ** of the vectors **d** and **y,** or the model error, can be estimated through a mean squared error (MSE)

(9) $$E\left(w,\;b\right)\;=\;\frac{1}{l}\sum_{q\;=\;1}^{l}{\left(d_{q}\;-\;y_{q}\right)}^{2},$$

where *l* is the number of elements of the error vector **δ**. By minimizing MSE during the ANN learning process, the weights and biases are gradually adapted to a suitably selected training dataset. ANN adaptation to training datasets starts with a random initialization of the weights and biases, and it stops when a pre-determined maximum number of iterations is reached or when the MSE function is minimized to the desired target value. The task of ANN training is therefore to find the correct weights and biases for the training dataset, which represent the unknown coefficients of a suitable analytical model of experimental data [50].

For the network training, the Levenberg–Marquardt error back-propagation minimization algorithm with an iterative scheme of weights and biases modification was used in the form

(10) $$z_{q\;+\;1}-\;z_{q}\;=\;{\left(J_{q}^{T}J_{q}\;+\;\lambda_{q}I\right)}^{-1}J_{q}^{T}E_{q}\;,$$

where *z* represents the weights *w* and biases *b* of the network, **J^T^***_q_* **J***_q_* is the Hessian matrix approximation for optimization of the ANN by the nonlinear least-squares method, **J***_q_* is the Jacobian matrix with respect to the *w* and *b*, **I** is the identity matrix, *λ_q_* is the learning parameter, and *E_q_* the error function of MSE. The symbol **^T^** is the **J***_q_* matrix transposition [51].

If the ANN adaptation to the training data is too accurate, the found model describes the given experimental data very well, but its predictive ability may be very low, which is referred to as over-fitting of the network. To prevent this, during the training of the ANN, the MSE evolution is also monitored in a validation dataset consisting of the data that are not contained in the training dataset, but which come from the same distribution. If the training data error decreases while the validation data error continually increases, training of the network will be prematurely terminated. The weights and biases, at which the MSE of validation dataset is minimal, will be then considered to be correct. The over-fitting of the ANN is related to a too high number of neurons, while the under-fitting (i.e., the inability of the network to describe more significant nonlinearities of experimental data) in the hidden layer has too low a number of neurons in the hidden layer, the validation of the ANN tunes its architecture by finding an optimal number of hidden neurons and at the same time tests the performance of the found ANN model during the prediction of the experimental results based on the unknown (validation) input-target patterns [52].

The testing dataset contains input-target patterns designed to test the ANN generalization ability for the data that is never learned. The testing set error is also used to compare the performance of different models at the prediction of new experimental results, which are unknown to the network. During the process of training and validation, the evolution of the testing error is also monitored. If the error value in the testing dataset reaches its minimum at a significantly different number of learning algorithm iterations than the validation set error, it is then highly probable that division of the experimental data was poor and it must be modified [53].

## 3. Results

### 3.1. Results of Tensile Tests

**Figure 2:**
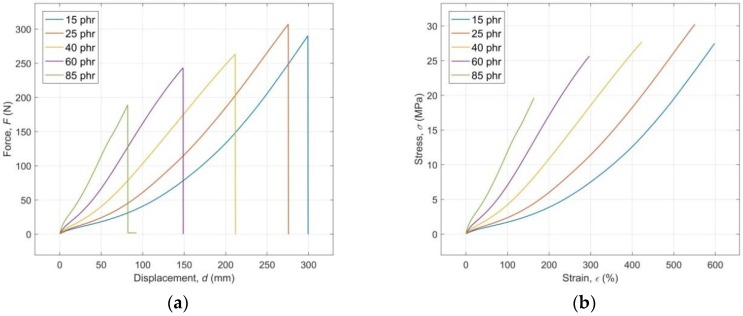
(**a**) Experimental average force versus displacement curves; (**b**) Computed average engineering stress-strain curves.

The results of uniaxial tensile tests for the investigated RB with different content of CB ranging from 15 to 85 phr, in the form of average force (*F*) versus displacement (*d*) curves, are presented in Figure 2a. The engineering stress-strain curves, computed from these experimental data, are presented in Figure 2b, which shows that with the increasing tensile strain *ε* the tensile stress *σ* grows in a non-linear but continuous manner, while the slope of the stress increase (modulus of elasticity) grows with the increasing CB content *c* in the RB. The reinforcing effect of the CB fillers is clearly visible. The nonlinear character of the stress-strain curves follows from molecular arrangements and strain-induced crystallization of CB filled RB [32].

Regression analysis of the maximum values of stress-strain curves has shown that tensile stress-at-break *σ*_b_ is a non-monotonically decreasing quadratic function, whereas tensile strain at break *ε*_b_ is a monotonically decreasing quadratic function of CB contents in the RB. This means that with an increasing CB content in RB the resulting material becomes more brittle and breaks at a smaller deformation. We can also observe that the stress-at-break after reaching its maximum at 25 phr of CB in the mixture is decreasing more and more sharply.

**Figure 3:**
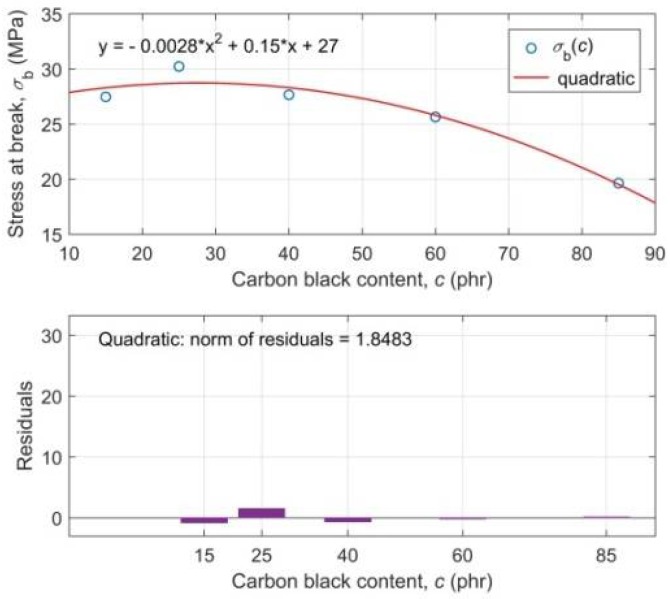
Regression analysis of the experimental dependence of the tensile stress-at-break *σ*_b_ on the content of carbon black *c* in the rubber blend (top plot); Residuals of parametric fitting of the experimental data *σ*_b_(*c*) with the estimation of goodness-of-fit (lower plot).

**Figure 4:**
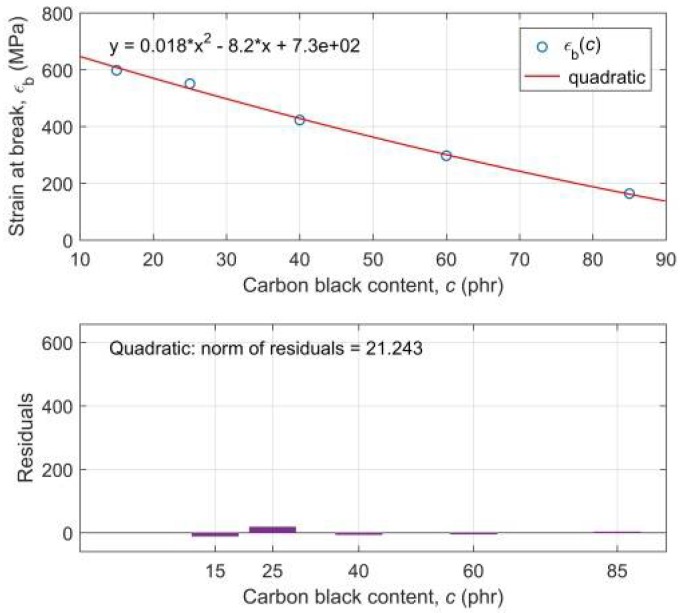
Regression analysis of the experimental dependence of the tensile strain at break *ε*_b_ on the carbon black content *c* in the rubber blend (top plot); Residuals of parametric fitting of the experimental data *ε*_b_(*c*) with the estimation of goodness-of-fit (lower plot).

Regression models of experimental functional dependencies *σ*_b_(*c*) and *ε*_b_(*c*), along with the residuals of parametric fitting of experimental data and the evaluation of the goodness-of-fit by the parameter “norm of residuals”, are presented in Figure 3 and Figure 4, respectively. A practically random distribution of residuals and very low values of the “norm of residuals” document a high degree of correlation of regression curves with experimental ones [54].

The identified stress-strain behavior of the investigated RB is typical for all particulate composites with a hyper-elastic matrix in a rubbery state, in which, in general, the addition of an active particulate filler following an increase in stiffness of the resulting material enhances its modulus of elasticity, reduces ductility, and from a certain amount of filler in the matrix it also reduces its strength [55].

The regression model of the functional dependence *ε*_b_(*c*) was later used at simulation of the ANN with the modelled input data, not known to the ANN, which made it possible to verify the generalization capabilities of the found ANN model of experimental data *σ*(*ε,c*) and it dramatically increased its practical use.

The mechanism, by which CB influences the physical properties and mechanical characteristics of RB, such as strength, elasticity, plasticity, toughness, tensile modulus, stress and strain at break, and a whole host of others, is somewhat complicated and it has been the subject of numerous studies. Their extensive overview can be found, for example, in [56]. Since the aim of the present paper is the prediction of the tensile response of RB with different CB contents to the mechanical tension load with the use of an ANN, the mechanism, by which different quantities and types of CB affect their physical properties and mechanical behavior or a more detailed analysis of the obtained stress-strain curves, are not addressed.

### 3.2. Pre-Processing of Experimental Data

Only four out of five available tensile curves with CB content of 15, 25, 60, and 85 phr were used for training of the proposed network, for its validation and testing of the found ANN model. The fifth curve was simulated by the already trained ANN and the results were then compared with the available experimental data (40 phr), which made it possible to test the generalization capabilities of the model. However, the creation of the ANN model of the experimental results required a special pre-processing of experimental data, described below.

The input and target data of the proposed FF-BP-ANN, stored in the parameters *Inputs* and *Targets*, were inserted into two matrices in the form **Inputs** = [**ε***;* **c**] and **Targets** = [**σ**]. The matrix **Inputs** consists of a row vector **c** of the CB contents *c^j^* in the RB and a row vector **ε** with the average input values of the tensile tests *ε^j^_i_*, while the matrix **Targets** consists only of a row vector **σ** of the average tensile test outputs *σ^j^_i_* at strains *ε^j^_i_* and CB contents *c^j^*, where *i* = 1, 2, 3, …, *n* and *j* = 1, 2, 3, …, *m*, while *n* is the number of samples of *ε_i_* (identical to the number of samples of *σ_i_*) for each *c^j^*, and *m* is the number of *c^j^*. Therefore, the parameters *Inputs* and *Targets* are presented in a matrix form as

(11) $$Inputs\;=\;\left(\begin{array}{l} c^{1}\;c^{1}\;\ldots \;c^{1}\;c^{2}\;c^{2}\;\ldots \;c^{2}\;\ldots \;c^{m}\;c^{m}\;\ldots \;c^{m}\\ \varepsilon_{1}^{1}\;\varepsilon_{2}^{1}\;\ldots \;\varepsilon_{n_{1}}^{1}\;\varepsilon_{1}^{2}\;\varepsilon_{2}^{2}\;\ldots \;\varepsilon_{n_{2}}^{2}\;\ldots \;\varepsilon_{1}^{m}\;\varepsilon_{2}^{m}\;\ldots \;\varepsilon_{n_{m}}^{m} \end{array}\right)$$

and

(12) $$Targets\;=\;\left(\sigma_{1}^{1}\;\sigma_{2}^{1}\;\ldots \;\sigma_{n_{1}}^{1}\;\sigma_{1}^{2}\;\sigma_{2}^{2}\;\ldots \;\sigma_{n_{2}}^{2}\;\ldots \;\sigma_{1}^{m}\;\sigma_{2}^{m}\;\ldots \;\sigma_{n_{m}}^{m}\right)$$

with dimensions

(13) $$Inputs_{\mathrm{size}}=\;2\;\times \;\sum_{j\;=\;1}^{m}n_{j}$$

and

(14) $$Targets_{\mathrm{size}}=\;1\;\times \;\sum_{j\;=\;1}^{m}n_{j}.$$

Since the input and output values of the tensile tests, as well as the CB content in the RB are measured in different units, and the difference between them is relatively very high, the parameters *Inputs* and *Targets* were normalized to the interval [−1,1] according to the formula

(15) $$y\;=\;\left(y_{\max}\;-\;y_{\min\;}\right)\frac{x\;-\;x_{\min\;}}{x_{\max}\;-\;x_{\min\;}}\;+\;y_{\min},$$

where *x* represents the original data before the normalization, *x*_min_ and *x*_max_ are their minimal and maximal values before the normalization, *y* represents the data after the normalization while *y*_min_ and *y*_max_ are their minimal and maximal values after the normalization representing −1 and 1, respectively [57]. It is exactly such normalization of the input and target data, which is at the applied sigmoidal transfer function of the hidden layer able to ensure the rapid and accurate convergence of the ANN output data to the values of the analyzed experimental data [22]. The normalized input and target data, with the total number of 2 × 9664 and 9664 samples, respectively, were randomly divided into training, validation, and testing datasets in a ratio of 0.70:0.15:0.15 [23], with the number of 2 × 6764, 1450 and 1450 samples, respectively. The training, validation, and testing datasets were subsequently used in the below-described optimization of the predictive ANN model.

### 3.3. Optimization of the ANN Model

The optimization of the predictive ANN model consists, in general, in such a setting of the ANN parameters, in which the difference between the output and target data is minimal, and at the same time the generalization capability of the ANN model is maximal [58]. The multilayer FF-BP-ANN, with the architecture shown in Figure 1, provides for *σ*(*ε*,*c*) optimal results with a minimal mean squared error of MSE, maximal linear correlation coefficient *R* between the ANN output and target data for all three datasets (training, validation and testing) simultaneously and with minimal differences between the experimental and simulated data, produced by the trained network with unknown input data, at six neurons in its hidden layer. Since the ANN adaption begins with random values of weights and biases, this causes each solution found in the process of its optimization to be totally different from all others and it does not provide any guarantee that it is the best solution [25]. Training, validation, and testing was repeated until the network simulation with unknown input data (for a specimen containing 40 phr of CB) did not provide the smallest difference between its output and target data corresponding to the applied input data. Considering that the input layer has two, while the output layer has only one neuron, the optimal architecture of the multilayer FF-BP-ANN for modelling and predicting the tensile curves of the investigated RB with different CB contents can be presented in a numerical form as 2-6-1 [22].

### 3.4. Analysis of the ANN Model

Neural networks usually do not allow a calculation of standard statistical parameters and diagnostics that are commonly used for the evaluation of the results of a regression analysis of experimental data [22]. Therefore, other methods were used to assess the goodness of the ANN model in detail, namely performance, training state, regression, and model error plots, as well as ANN error histogram [36].

**Figure 5:**
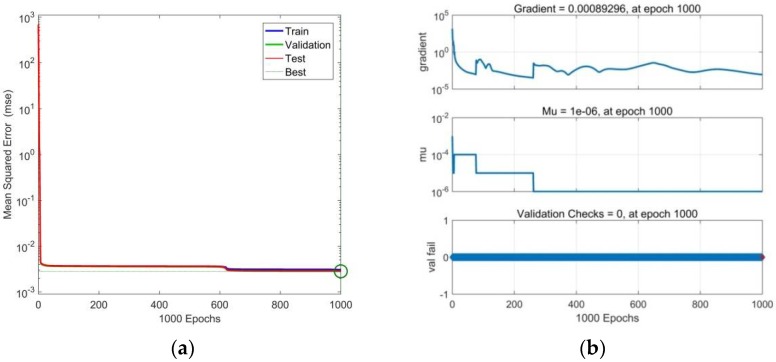
(**a**) Performance plots of the neural network model; (**b**) Training state plots of neural network model.

The performance plot, which shows how the MSE function is minimized during the training process, is shown in Figure 5a. It is clear from this figure that the error of training, validation, and testing data during the learning continuously decreases, while all three curves have practically the same character without any significant traces of over-fitting [30]. The best validation performance of the ANN model (marked by a green circle) occurs in the last iteration cycle, with the associated final MSE value, which is relatively small for all three datasets and indicates the obtained results are very good for both the approximation and for the prediction of the experimental values of all four analyzed functional dependencies *σ*(*ε*,*c*).

The process of the network learning till the moment of its premature termination can be traced also on the training state plots shown in Figure 5b, which document that the Levenberg–Marquardt gradient of the learning algorithm, as well as the network validation error mu, show a decreasing trend, with the number of iterations (epochs), in which the MSE of validation data has increased its expression as a parameter value fail, is zero. From the mentioned training state plots, it is clear that the training process, during which the network is continuously being learned is highly stable and it shows no signs of an over-fitting due to the high number of hidden neurons or samples of input data [32].

**Figure 6:**
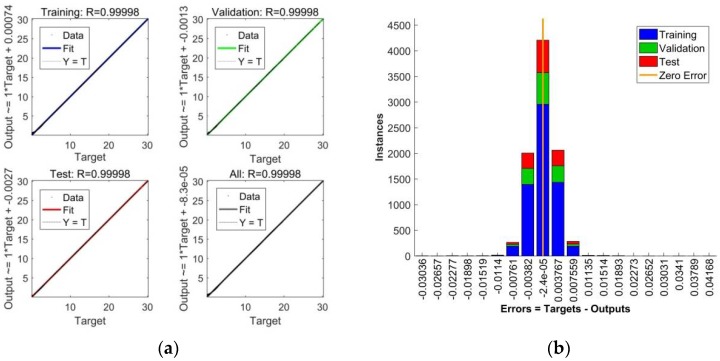
(**a**) Linear regression plots for training, validation, testing, and all data used in the optimization process; (**b**) Error histogram for training, validation and testing data.

The degree of correlation between the output and target data is usually expressed by the linear correlation coefficient

(16) $$R\;=\;\frac{Cov\left(d,\;y\right)}{s_{d}s_{y}}$$

for the training, validation, and testing datasets, as well as for all experimental data used for an optimization of the ANN model. The symbol *Cov(d,y)* is the labelled covariance of the output and target data, the symbols *s*_d_ and *s*_y_ are their standard deviations, respectively [15]. The values of *R* coefficients are shown at the top part of the linear regression plots or fitness plots presented in Figure 6a. The left side of the fitness plots gives the linear fit line equation.

It is evident from the linear regression plots shown in Figure 6a, that practically all the data falls directly on the 45° line with the associated *R* value (slope) of 1, expressing the exact match between the ANN output (computed) and target (experimental) data [36], while the maximum data deviations can be seen at the beginning of the analyzed stress-strain curves.

The error histogram shown in Figure 6b documents that all errors of the ANN model are almost symmetrically distributed around the zero value and that they fall into a relatively narrow interval. A relatively small number of relatively small outliers in the left and right parts of the histogram, corresponding to the data scattering at the beginning of the linear regression plots, confirms the achievement of outstanding results in the approximation and prediction of the experimental values of all four analyzed functional dependencies *σ*(*ε*,*c*) [25].

**Figure 7:**
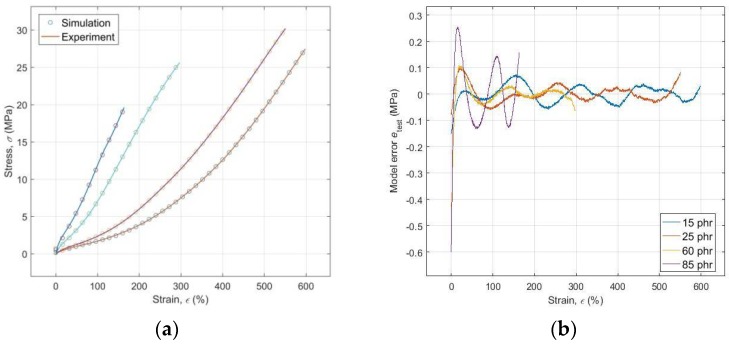
(**a**) Comparison of experimental and simulated data; (**b**) Model error plot.

Graphical comparison of the experimental data *σ*(*ε*,*c*) with the simulated data produced by the optimized ANN model of the trained FF-BP-ANN and the difference between them *e*_test_(*ε*,*c*), or the model error plot, are presented in Figure 7a,b, respectively. It is evident from these figures that the maximum error model, at a level of approximately 3% of the maximum value *σ*, is at a simulation of the tensile test results for the specimen of RB with a CB content of 85 phr, which represents its excellent performance both at the approximation and in the prediction of all four analyzed functional dependencies *σ(ε,c*).

### 3.5. Generalization Capabilities of the ANN Model

**Figure 8:**
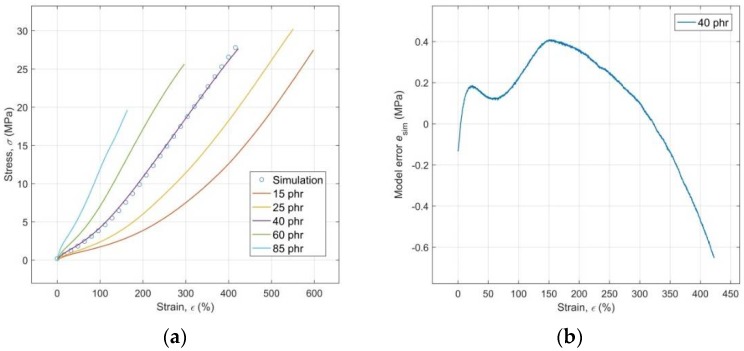
(**a**) Results of simulation of the optimized model with unknown input compared to the experimental stress-strain curves; (**b**) Model error plot of simulated data.

During an optimization of the ANN model, the experimental stress-strain curve for the specimen of RB with a CB content of 40 phr (excluded from the ANN training, validation and testing process) was used for an optimization of the generalization capabilities of the trained network at a prediction of *σ*(*ε*,*c*) based on unknown input values *ε* and *c*. The results of the simulation of the optimized ANN model with these unknown input data compared to the experimental stress-strain curves are shown in Figure 8a. Both graphs shown in Figure 8a,b document the achievement of an excellent performance of the optimized ANN model also at the prediction of tensile stress of the investigated RB with an unknown CB content and the corresponding unknown tensile strain of test specimen, with a maximum error of the model *e*_sim_ approximately 2.4% of the maximal value *σ* (Figure 8b).

### 3.6. Simulation of the ANN Model Based on Modeled Inputs

**Figure 9:**
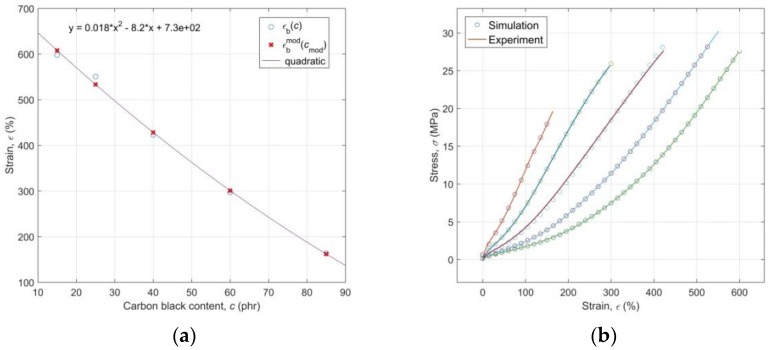
(**a**) Selection of the *ε*_b_^mod^ values for simulation of the optimized model with modeled input data; (**b**) Comparison of the experimental stress-strain curves with the curves simulated by a model with modeled input data.

From a practical point of view, it is interesting to find out whether the optimized ANN model, described in detailed and analyzed above, will be able to predict—with a sufficiently high degree of reliability—the course of the stress-strain curves at any value of strains and CB contents in the investigated RB that are unknown to the network. To verify the predictive abilities of the model under the above-mentioned conditions, the following original simulation procedure of the trained network with modeled input data was made:

An identified quadratic regression model (Figure 4) in the form of function

(17) $$\varepsilon_{b}\left(c\right)\;=\;0.018c^{2}\;-\;8.2c\;+\;730$$

makes it possible for any CB content *c*_mod_ in the investigated RB to determine a corresponding value of strain at break *ε*_b_^mod^ (Figure 9a), which determines the length of an input matrix of the optimized model, and afterwards to approximate the values of the network input vectors **ε_mod_** and **c_mod_** through the relations

(18) $$\varepsilon_{mod}\;=\;0\;:\;step\;:\;\varepsilon_{b}^{\mathrm{mod}}$$

and

(19) $$length\left(c_{mod}\right)\;=\;length\left(\varepsilon_{mod}\right)\;,$$

so the input parameter for simulation of the trained network gets the form

(20) $$Inputs_{\mathrm{mod}}\;=\;\left(\begin{array}{l} c_{\mathrm{mod}}^{1}\;c_{\mathrm{mod}}^{2}\;c_{\mathrm{mod}}^{3}\;c_{\mathrm{mod}}^{4}\;\ldots \;c_{\mathrm{mod}}^{\varepsilon_{b}^{\mathrm{mod}}}\\ 0\;1\cdot step\;2\cdot step\;3\cdot step\;\ldots \;\varepsilon_{b}^{\mathrm{mod}} \end{array}\right)\;,$$

where length(.) measures the length of the relevant input vector and *step* is a spacing between its individual elements. It is evident from the results of the network simulation with the thus modelled inputs of **ε_mod_** and **c_mod_** that the optimized ANN model is capable of the successful prediction of stress-strain behavior of the investigated RB with any content of CB within the whole range from 15 to 85 phr (Figure 9b) and with the corresponding strains without the necessity of realizing the time consuming and costly process of the mixing of RB with the various content of CB, the manufacture of testing specimens, and the subsequent testing of their response to mechanical loading with the use of tensile tests. At this point, it is important to emphasize that the area of the excellent predictive performance of the ANN model is essentially limited to just the width of the interval of the input-target patterns learned by the network, which is a consequence of the relatively poor extrapolation capabilities of ANNs in general (as opposed to their excellent interpolation performance) [59]. Figure 9a shows the manner of selection of the approximated values *ε*_b_^mod^ based on the regression model *ε*_b_(*c*) for the simulation of the optimized ANN model with the modeled input data in a way that its output could be compared with all five experimental stress-strain curves that were available (Figure 9b).

Since the experimental and simulated stress-strain curves are created from the data produced at different frequencies (or at different values of the parameter *step*), and they have different lengths, the construction of the error plot for the ANN model with modeled inputs is impossible. Its performance at the prediction of the tensile test results with unknown, modeled input data was therefore assessed by an estimate of error of prediction of the strain at break *ε*_b_, expressing the total ductility of RB, stress-at-break *σ*_b_, which is a significant strength characteristic of the materials, and the M100 tensile modulus measured at 100 percent strain, characterizing the stiffness of the elastomers [60]. The prediction error of the respective mechanical characteristics *X* was calculated according to the relationship [19]

(21) $$Error_{X}\;=\;\left|\frac{100\;\left(X_{\exp}\;-\;X_{\mathrm{sim}}\right)}{X_{\exp}}\right|\;,$$

where the index _exp_ is related to the experimental data and the index _sim_ to the simulated data produced by the optimized 2-6-1 FF-BP-ANN model with unknown modeled input data. Error estimation Error_x_ of the respective mechanical characteristics X for the investigated RB with different CB contents is presented in Table 1, which reveals an excellent consistency between the experimental and predicted tensile test results.

As we have already mentioned in the introduction of our work, there are several classes of phenomenological approaches to develop a constitutive stress—strain relationship that can fit the experimental results for hyperelastic materials [14]. These constitutive models, however, require the identification of the unknown coefficients contained therein from the relevant experimental data. Similarly, standard regression models only provide a description of real experimental stress—strain curves, and their prediction under new conditions (with different material compositions and other tensile loads) is usually too time-consuming and often impossible. However, if we abandon the requirement to predict the full stress—strain curve, then the regression models can serve to predict tensile characteristics such as ε_b_, σ_b_, and whether the tensile modulus is at the given strain. On the other hand, the ANN modeling described above allows a highly efficient prediction of the entire tensile response of the tested material under different conditions without limitation of classical modeling.

## 4. Conclusions

In the present work, a multilayer artificial neural network approach has been employed for prediction of the mechanical behavior of a rubber blend with different contents of carbon black under uniaxial tensile loading. A 2-6-1 feed-forward back-propagation artificial neural network model, with an experimental carbon black content and strain values as model inputs and corresponding experimental stress values as model targets, trained by the error back-propagation Levenberg–Marquardt optimization algorithm, has been developed to predict the engineering tensile stress-strain curves of a rubber blend with content of carbon black ranges from 15 to 85 parts of carbon black per hundred parts of rubber. A novel procedure has been created for the simulation of the optimized artificial neural network model with modeled input data generated by a regression model of experimental strain at break dependence on the carbon black content in the investigated blend. The error of prediction of the strain at break and stress at break, as well as of the M100 tensile modulus measured at 100 percent strain was estimated. Comparison of experimental stress-strain curves with the results of all simulations has demonstrated an excellent consistency between the experimental and simulated data over their entire range. The results achieved in the presented work justify an assumption of the authors that the created artificial neural network model can find a wide application in the efficient design of the composition of rubber blends for numerous applications in different areas of industrial practice.

## Figures and Tables

**Table 1:** Error estimation of mechanical characteristics X for modeled input model data.

Error_x_	12 phr	25 phr	40 phr	60 phr	85 phr
*ε*_b_ (%)	0.33	5.92	0.67	0.97	2.38
*σ*_b_ (%)	0.44	8.82	1.57	1.08	3.04
M100 (%)	0.52	2.36	5.45	0.35	0.85
